# SAR minimum entropy autofocusing based on Prewitt operator

**DOI:** 10.1371/journal.pone.0276051

**Published:** 2023-02-10

**Authors:** Xiaoze Hou, Yanheng Ma

**Affiliations:** Army Engineering University, Shijiazhuang, China; University of Isfahan, ISLAMIC REPUBLIC OF IRAN

## Abstract

Current autofocus algorithms utilizing image criteria impose a significant computational burden. Therefore, this paper proposes a computationally efficient autofocus algorithm combined with SAR image feature points, employing the Prewitt operator to obtain the SAR image features. The range cell with the number of feature points in the front row as the input of the autofocus method to perform motion error estimation and compensation on SAR imagery. Our method’s key feature is to optimize the selection criteria of range cells by acquiring the feature points of SAR images,reduces the number of input range cell,reduce the computational complexity of the autofocus algorithm and ultimately enhance the focusing effect of SAR images. Trials involving simulation and measured data demonstrate the effectiveness of the developed method.

## 1. Introduction

Synthetic aperture radar(SAR) is a high-resolution imaging radar that can obtain high-resolution radar images under any weather condition and light conditions [1–4]. These advantages of SAR make it widely used in industry and agriculture.The rise of research content in deep learning in several years, which fuses deep learning technology with SAR imaging target identification research, has enhanced the readability of SAR images, it can be said that SAR has a much broader application scenario [5–7].

With the wide use of new small and medium-sized platforms such as unmanned aerial vehicles, the research of airborne SAR system has attracted extensive attention. Compared with spaceborne SAR system, the application of airborne SAR system is more flexible. However, because the carrier is inevitably affected by unstable airflow in the air, the movement track deviates from the ideal track. As W.G. Carrara, an expert in SAR, said, "Sport is the basis of SAR and the root cause of problems.". The motion errors caused by these non ideal motions have a huge impact on the radar echo phase. If the above errors are not compensated, the SAR imaging quality will be serious.

For Airborne SAR systems, it is impossible to carry heavier and bulky inertial navigation devices due to the limited precision of some inertial navigation devices and the load limitations of some carrier. Therefore, data-driven autofocusing techniques are commonly used to compensate for phase errors [8–10].

Common autofocuse algorithms mainly include MD(Map Dridt), MEA(Minimum Entropy Autofocus), PGA(Phase Gradient Autofocus), etc. Compared with the PGA and other autofocus algorithms based on specific scattered points, the image-criteria-based autofocus algorithm has a broader range of applications because it does not rely on strong scattering points [11]. Among current algorithms, the MEA [12], a classic image autofocus algorithm,has received extensive attention [13–16].

The MEA algorithm considers image entropy as the cost function to estimate the motion error and compensates the defocused image by optimizing the cost function. However, during MEA’s iterative process, the calculation complexity of the objective function’s entropy is significant, and the convergence speed is slow, leading to the algorithm’s low efficiency [17]. In order to improve the operational efficiency of MEA method, it is mentioned in [18,19] that the weight of strong scattering points in the whole image is improved by the minimum variance criterion based on least square estimation. These methods can improve the convergence accuracy and speed of the algorithm. Nevertheless, some range cell do not extract helpful information in complex SAR images. Therefore, it is possible to select some range cell containing more phase error information of the imaging scene, significantly reducing the calculation complexity of the autofocus algorithm and improving its efficiency.

S. Wei et al [20] exploited the range cell, whose energy is located in the first 5%~20%, to compensate for this computational burden as the input to the autofocus algorithm. However, when the strong reflection points in the scene are concentrated, the range cell are relatively concentrated.Ran, Lei, et al [21] suggests that the SAR phase error often reflects range spatiality, especially in wide scenes. If the range units substituted for MEA algorithm are too centralized in the range dimension, the calculated error phase can not achieve high-precision motion compensation for the whole image, resulting in the degradation of SAR image quality. Additionally, the [22] mentioned method for SAR image autofocus still requires improving the computational efficiency and focusing accuracy.

Aiming at the above problems, this paper proposes a Minimum Entropy Autofocus algorithm based on the Prewitt operator(PMEA). Specifically, our method uses the Prewitt operator to extract the feature points in the defocused SAR image and utilizes the range cell with the number of feature points in the front row as the input to the MEA,realized the reduction of the amount of calculation and the improvement of the efficiency of the algorithm.Furthermore, our measured data and simulation results reveal that when using the range cell with more feature points as the input to the MEA,the focusing effect of the MEA improve. Finally, when the strong scattering points are concentrated in the scene, employing PMEA effectively improves the focusing effect of the SAR image. Overall, the simulation and measured data show that the proposed method effectively compensates the motion error affording a small computational complexity,fast convergence speed,appealing SAR image focusing effect,and high error compensation accuracy.

## 2. PMEA based on Prewitt operator

### 2.1 Minimum entropy autofocus method

The MEA algorithm is a classic autofocus algorithm based on image criteria [12]. Image entropy is describes the average information content of the image source.The MEA algorithm believes that the smaller the entropy of the SAR image, the better the focusing quality. The MEA algorithm starts from the defocused SAR complex image data, takes the image entropy as the cost function, and compensates the azimuthal phase error of the echo signal to reduce the image entropy.

The Shannon entropy of a SAR image I is defined as:

$$E(I)=-\sum_{q=0}^{Na-1}\sum_{k=0}^{Nr-1}D(q,k)\cdot \ln\left[D(q,k)\right]$$ (1)

Among them, *q* is the azimuth pulse index, *Na* is the number of azimuth pulses; *k* is the range cell index, and Nr is the number of range cell.

*D(q*,*k)* is the scattering intensity density of the image,

$$D\left(q,k\right)=\frac{{\left|I(q,k)\right|}^{2}}{S(I)}$$ (2)

In the Eq (2), *I*(*q*,*k*) is the amplitude of each scattering unit in the imaging scene. $S(I)=\sum_{q=0}^{Na-1}\sum_{k=0}^{Nr-1}{\left|I(q,k)\right|}^{2}$ is the total energy of the image, which is a constant.

The minimum entropy method starts from the complex image domain, and the data is transformed into the range compressed phase history domain through the azimuth inverse Fourier transform., if there are *N* scattering points in the kth range cell, the signal model for the kth range cell containing the phase error can be expressed as:

$$e(m,k)=\sum_{N=1}^{N}\sigma_{n}\exp\left\{j[2\pi f_{n}m+\phi_{e}(m)\right]\}$$ (3)

In the Eq (3), *m* is the azimuth pulse index; *k* is the index of the range cell; *f*_*n*_ is the doppler frequency of the scattering point in the kth range cell; *σ*_*n*_ is the backscattering coefficient of the *n*th scattering point. *ϕ*_*e*_(*m*) represents the phase error due to nonideal motion of the carrier.

The error phase estimated by the minimum entropy method is:

$${\hat{\phi }}_{e}(m)=diag[\hat{\phi }(0),\hat{\phi }(1),\cdots ,\;\hat{\phi }(Na-1)]$$ (4)

The solution process of the ${\hat{\phi }}_{e}(m)$ can be referred to reference [20].

The echo signal of the kth cell after phase compensation can be expressed as:

$$e(m,k)=\sum_{N=0}^{N-1}\sigma_{n}\exp\{j[2\pi f_{n}m+\phi_{e}(m)-\phi_{e}\hat{(}m)]\}$$ (5)

When the ${\hat{\phi }}_{e}=\phi_{e}$, the image was considered fully focused at the time.

**Fig 1:**
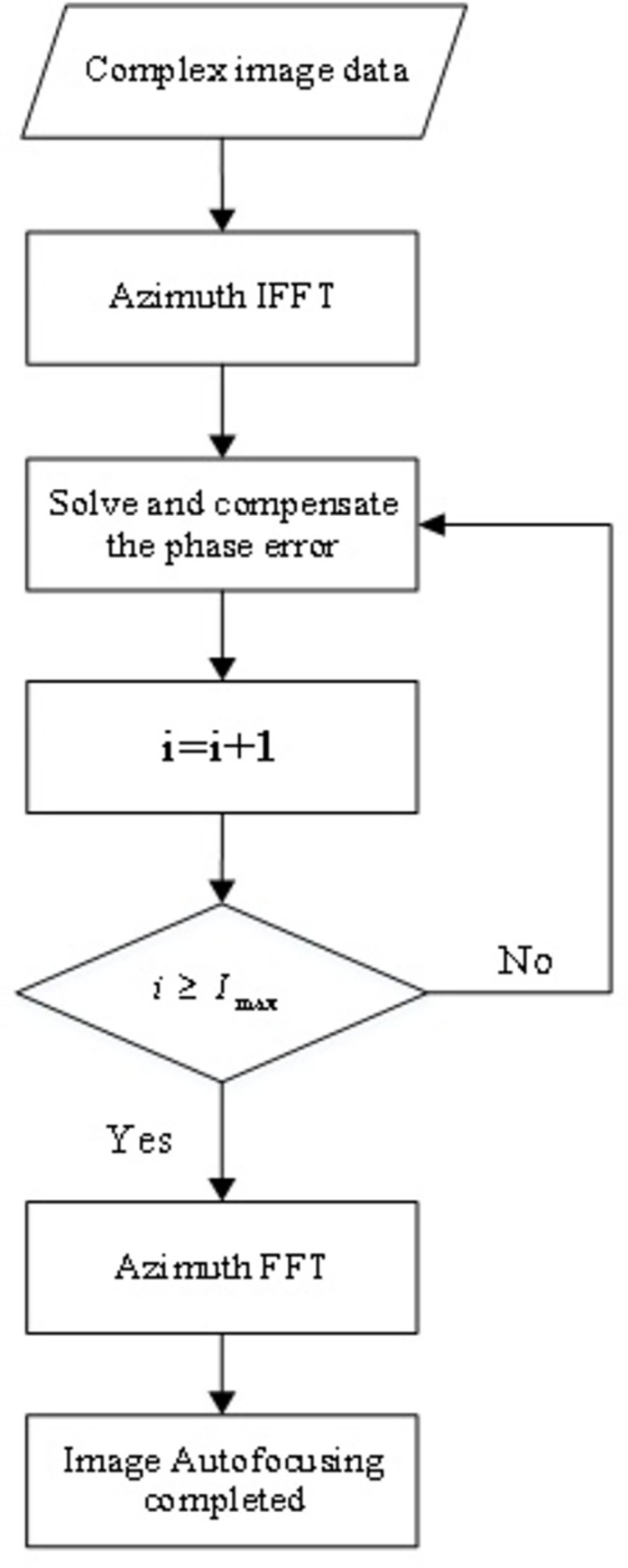
The flow chart of MEA algorithm.

The flow chart of MEA algorithm is shown in Fig 1. The iteration starts with i *i* = 0,the number of iterations *I*_max_ can be selected according to the specific SAR data:

### 2.2 Prewitt operator to extract image features points

The MEA algorithms can compensate for various phase errors in SAR images. Compared with PGA, it does not rely on strong scattering points and therefore has broader applicability. However, MEA suffers from slow convergence speed and algorithm operation efficiency due to the phase error searching process, the numerous entropy calculations in the objective function. Nevertheless, some range cell do not extract helpful information in complex SAR images [20]. Therefore, it is possible to select some effective parts of the imaging scene, significantly reducing the calculation complexity of the autofocus algorithm and improving its efficiency. In order to reduce MEA’s computational complexity, several engineering applications utilize the energy in the first 5%~20% of the range cell as the autofocus algorithm’s input. However, when the strong scattering points are more concentrated in the scene, using this method makes the range elements substituted with the MEA more concentrated in the range direction, which affects the focusing accuracy of the self focusing algorithm.

Inspired by the choice of partial range cells as input to the MEA algorithm, this paper proposes a range cell selection method based on the Prewitt operator. This method firstly uses Prewitt operator to extract the feature points of the defocused SAR image, and then takes a part of the range cell with the number of feature points in the front row as the input of the MEA. It greatly reduces the amount of calculation, improves the efficiency and accuracy of motion compensation.

The range cell containing many of these feature points is used as the input of the MEA method ensures that the range cell substituted into the autofocus algorithm are evenly distributed in the range direction.On the other hand, range cell with many feature points often contain rich phase error information. The range units with more feature points can affect the entropy value of the image more. The MEA algorithm estimates phase error by reducing the image entropy by compensating the phase error of the echo signal at the cost of the image entropy.

Hence, selecting these range cell as the algorithm’s input, effectively improve the algorithm’s motion compensation accuracy and iteration speed. It is worth noting that compared with directly using the range cell with more considerable energy as the input of the MEA, PMEA optimizes the range cell selection process, reduces the number of input range cell, accelerates the algorithm’s convergence, and reduces the convergence time. Overall, PMEA reduces the computational complexity and improves the computational efficiency and motion compensation accuracy.

The Prewitt operator is a first-order differential operator used to extract the feature points of the input image [23]. It is shown that it is feasible to use Prewitt operator to identify feature points in SAR images [24,25]. Considering this paper, the Prewitt operator extracts the feature points of the SAR images.

The grayscale difference of the upper and lower and the left and right adjacent points of the SAR image pixel reaches an extreme value at the edge. For the horizontal and vertical gradients, we select the appropriate gradient magnitude formula according to the SAR image to calculate the gradient magnitude of each pixel point. At this time, the amplitude peak corresponds to the position of the edge point.

The Prewitt operators are:

$$W_{h}=\frac{1}{3}[\begin{array}{ccc} -1 & 0 & 1\\ -1 & 0 & 1\\ -1 & 0 & 1 \end{array}]\;W_{v}=\frac{1}{3}[\begin{array}{ccc} -1 & -1 & -1\\ 0 & 0 & 0\\ 1 & 1 & 1 \end{array}]$$ (6)

Assuming that the SAR image contains a pixel, the gradient template is used to convolve the SAR image, and the horizontal and vertical gradients of each pixel are:

$$\left\{ \begin{array}{l} G_{h}(m,n)=F(m,n)*W_{h}\\ G_{v}(m,n)=F(m,n)*W_{v} \end{array}\right.$$ (7)

*F*(*m*,*n*) represents the amplitude of the (*m*,*n*) pixel of SAR image.

The gradient magnitude formula according to the characteristics of the SAR image, the gradient amplitude formula selected in this paper is shown in Eq 8:

$$G(m,n)=|G_{h}(m,n)|+|G_{v}(m,n)|$$ (8)

The calculation result of the above Eq (8) is the gradient image, which represents the gradient of each pixel in the digital image. Then select an appropriate threshold T to binarize the above gradient image.The operation process is shown in Eq 9.


$$B\left(m,n\right)=\{\begin{array}{l} 1\;;\;G(m,n)\geq T\\ 0\;;\;G(m,n)<T \end{array}\}$$ (9)

The output of the above Eq (9) is a binarized image, and the pixel point of 1 is the step-shaped edge point, that is the feature point of the SAR image extracted by the Prewitt operator.

This paper, the Prewitt operator is used to select characteristic point scatterers from SAR images as the input of the MEA. In digital image processing, the Prewitt operator is usually used to obtain the feature points of the input image. There are several reasons for using the SAR image feature points obtained based on the Prewitt operator as the input of the minimum entropy algorithm.

1. When the strong scattering distribution in the scene is relatively concentrated, the PMEA method ensures that the range cell substituted into the autofocus algorithm are evenly distributed in the range direction.
2. Considering that some range elements do not contain useful information in complex SAR images, PMEA algorithm selects some effective range elements for error estimation, which significantly reduces the computational complexity. For the method of directly selecting the ranger cell with strong energy as the MEA iteration, the PMEA method needs less time to achieve convergence, and the final image focusing effect is better.
3. The MEA algorithm estimates phase error by reducing the image entropy by compensating the phase error of the echo signal at the cost of the image entropy. The range units with more feature points can affect the entropy value of the image more. Therefore, selecting these units as the input of MEA algorithm can improve the efficiency of the algorithm and the accuracy of algorithm error compensation.

### 2.3 PMEA algorithm steps

The main steps to improve the minimum entropy self-focusing method based on the Prewitt operator are as follows:

1. Prewitt operator is used to obtain the feature points of the defocused SAR image, and the feature points matrix of the defocused SAR image is obtained. The matrix is a binary matrix, where 1 represents the location of the feature points of the input SAR image and 0 represents the location of the non-feature points.
2. Summarize the columns of the eigenvalue matrix (bearing direction, range direction) and sort the results in descending order, with the records in the top 5%~20% of the size of the columns in the matrix.
3. The range cell with a large number of feature points found in the second step is used as the input of the MEA algorithm, and the phase error is corrected through multiple iterations.

**Fig 2:**
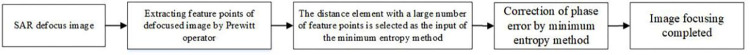
PMEA flow chart.

The flow chart of PMEA algorithm is shown in Fig 2.

## 3. Experiments and results

### 3.1 Simulation

The main waveform parameters are shown in Table 1.

**Table 1:** Main waveform parameters.

Carrier frequency	Bandwidth	Pulse width	Carrier flight speed
5×10^9^*hz*	2×10^8^*hz*	1.5×10^−6^*s*	100*m*/*s*

**Fig 3:**
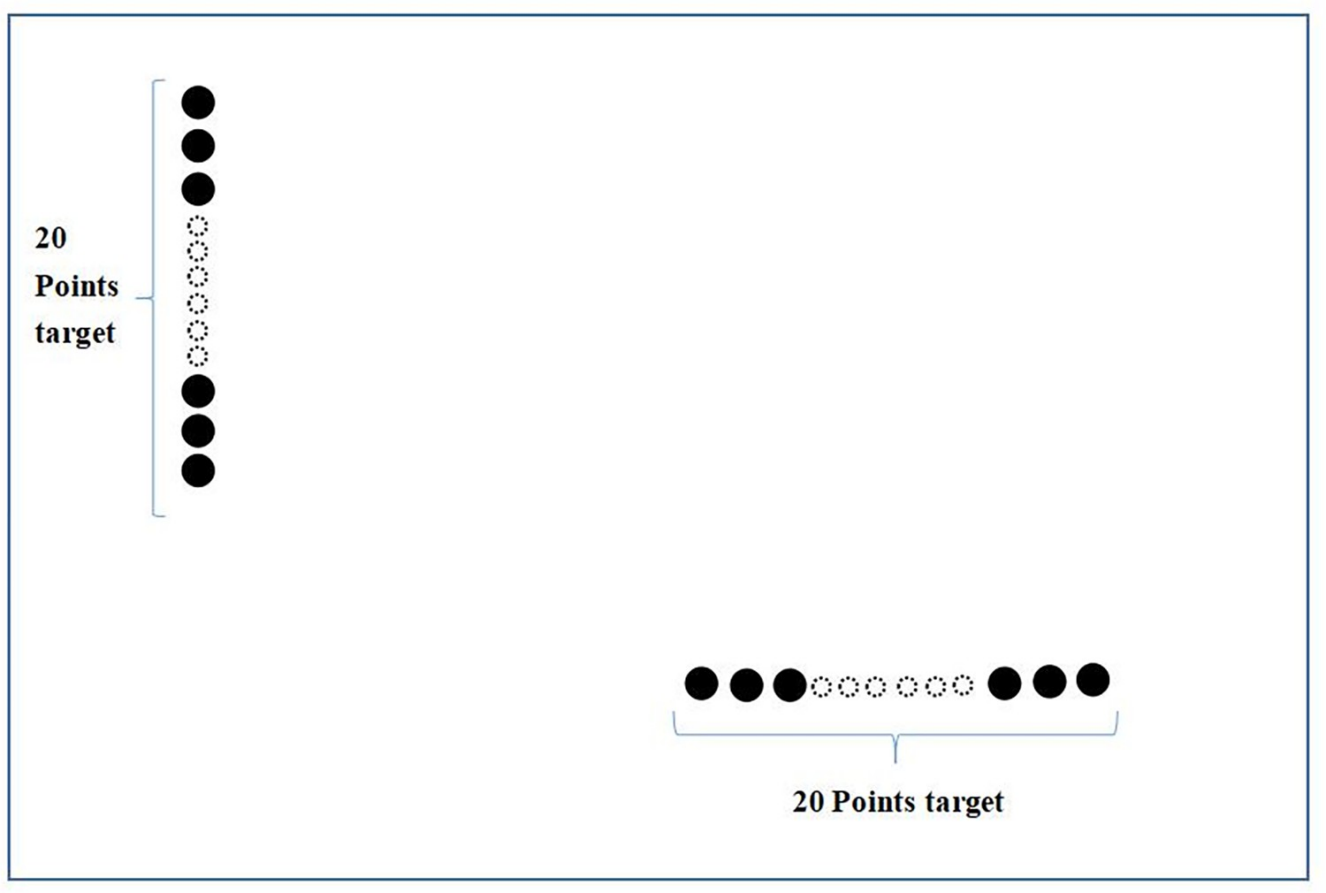
Schematic diagram of point target.

In order to verify the effectiveness of the motion compensation of the proposed algorithm,the scene point targets are set as shown in Fig 3, where in the backscattering coefficient of the 20 point targets vertically arranged at the upper left is set to 1. The backscattering coefficient of the 20 point targets arranged horizontally at the lower right is set to 1.5, which is used to simulate the strong scatterers with concentrated distribution in the scene.

**Fig 4:**
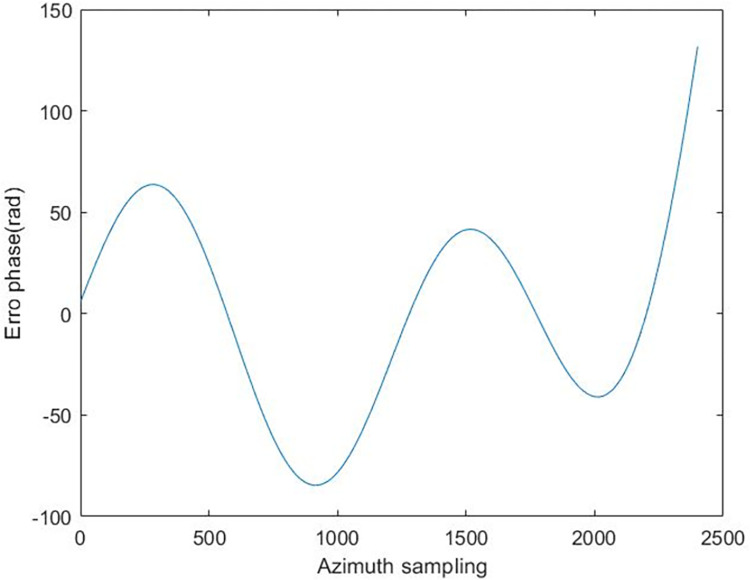
Error phase added to simulation image.

We add the phase error shown in Fig 4 to the already focused SAR image to simulate the motion error of the carrier aircraft in the air, and then take different autofocus algorithm performs motion error estimation and compensation for the above images.

The expression for the added phase error is

$$\overset{}{\phi }(m)=\exp\{j20\pi (\sin2\pi m)+m^{2}+m^{5}+0.5m^{6}\}$$ (10)

Where m is the azimuth pulse index.

Select PMEA, MCA and MEA to perform motion compensation on the above SAR data.In order to facilitate comparison, MCA and MEA algorithms select the top 5% of energy in all range cell in SAR echo data as the input of the algorithm, while PMEA algorithm selects the top 5% of feature points in all range cell as the input of the algorithm.

**Fig 5:**
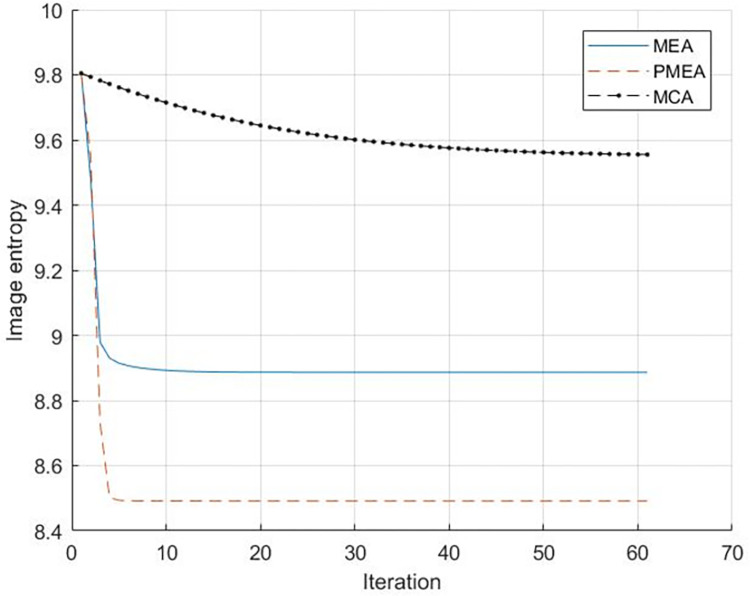
Entropy curve of simulation experiment.

The entropy of the image is used to evaluate the focusing effect of the algorithm. The number of iterations of each algorithm is 60.The entropy change curve of each algorithm in 60 iterations is shown in Fig 5.

**Fig 6:**
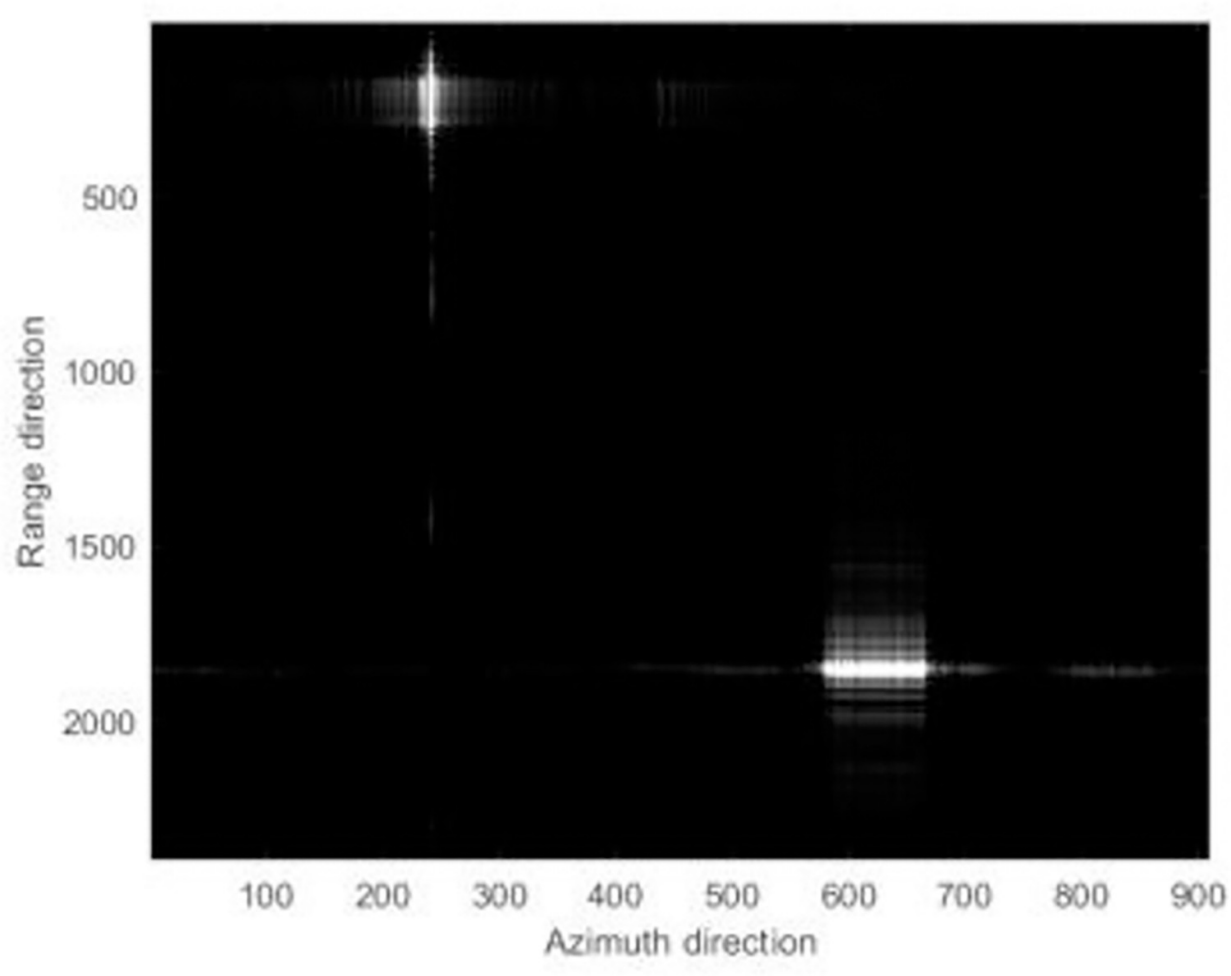
The simulation focused results.

The focusing results of different algorithms are shown in Fig 6.

**Fig 7:**
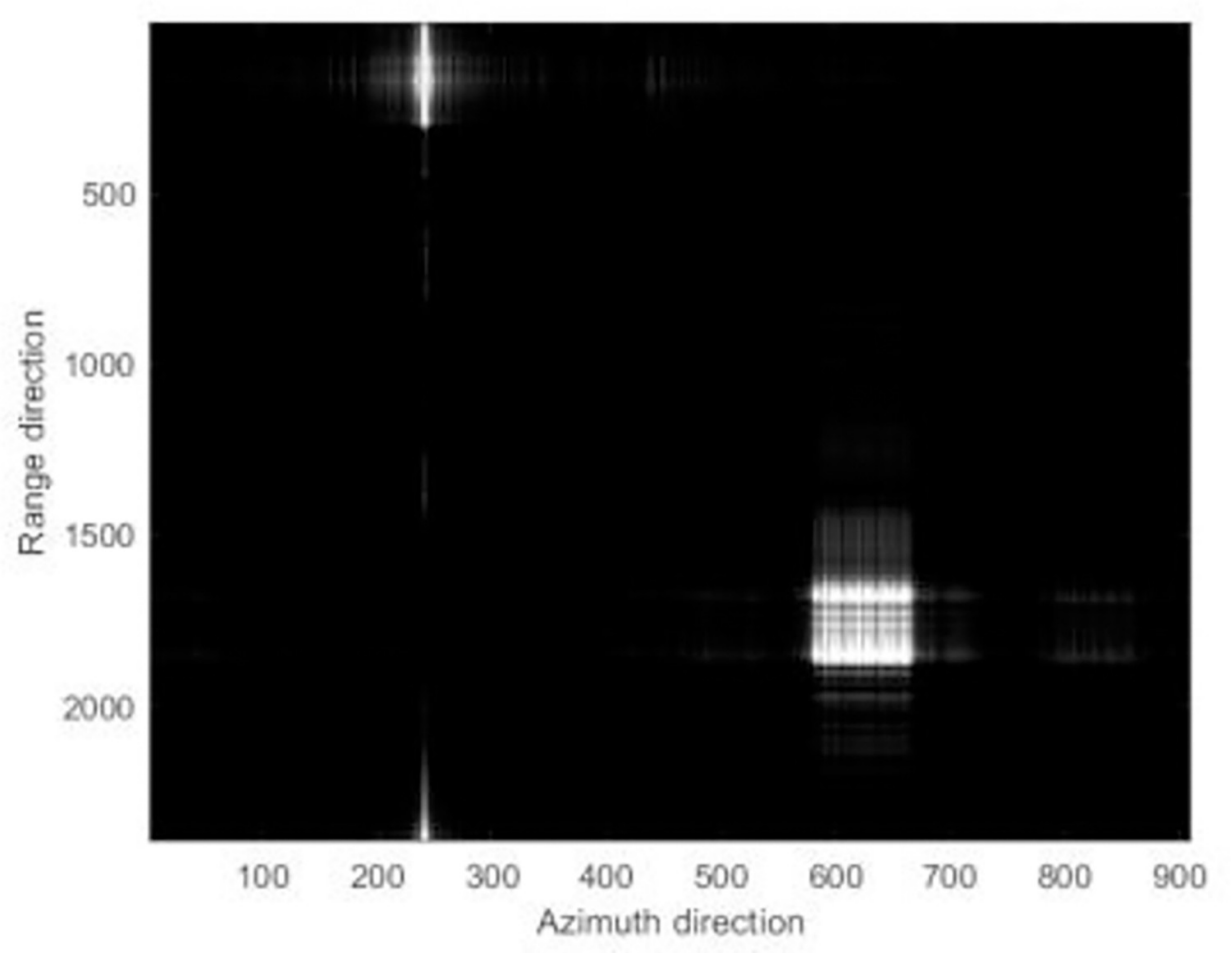
The simulation focused results.

**Fig 8:**
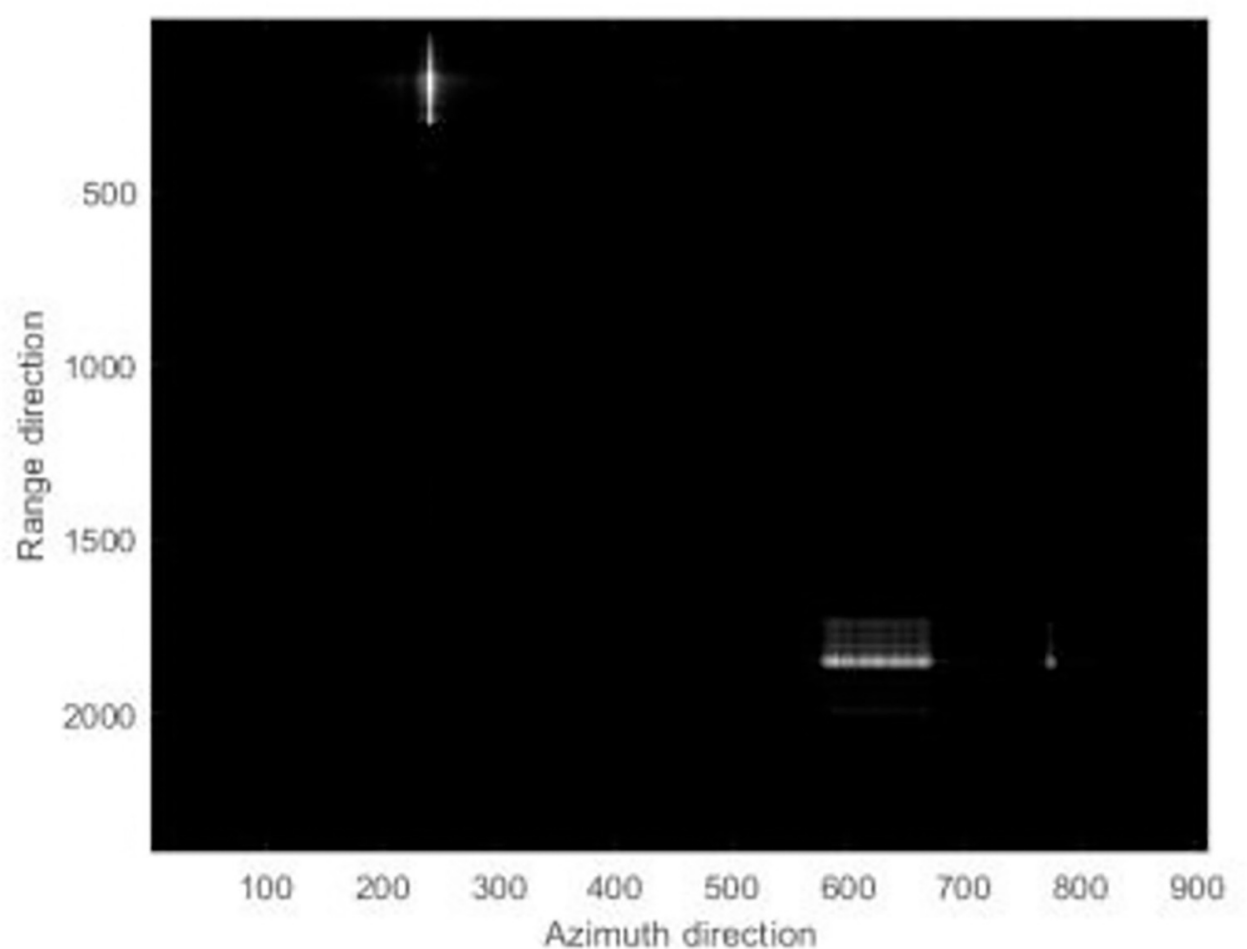
The simulation focused results.

Figs 6–8 reveals that the image presents a noticeable defocus effect when utilizing the MEA method and the MCA method.However, when the PMEA method is used, the focusing effect of the SAR image is better. Moreover, Fig 5 highlights that the PMEA method has the quickest convergence, the final image has the smallest entropy value and the best focusing effect. The final entropy value and time required for each algorithm to reach convergence are shown in Table 2.

**Table 2:** Comparison of simulation results.

	PMEA	MEA	MCA
**The final entropy value of the image**	8.50	8.89	9.56
**Convergence Average Time (s)**	0.65	1.10	3.99

Considering the MCA method, it requires the most time to converge, the entropy value of the final convergence is the lowest, and the MEA’s final image entropy value is higher than PMEA. This is because in the simulation scene, the distribution of the strong scatterers is relatively concentrated, and the energy of the range cell distributed by these strong scatterers is relatively large.MEA and MCA algorithm results in a concentrated distribution of the range cell substituted into the MEA. The MCA input is also concentrated in the entire range direction, increasing the convergence speed and the error, and presenting a low compensation accuracy. The PMEA method considers the above problems and selects as input a range cell that contains a large number of feature points, making the distribution of the substituted range cell more uniform. On the other hand, the range cell with many feature points contains rich phase errors. Hence, selecting these range cell as the algorithm’s input improves motion compensation accuracy and iteration speed. Therefore, the entropy value of the image’s final convergence is lower than the one using the MEA and MCA methods. Moreover, the average convergence time is also better than the competitor methods.

### 3.2 Measured data verification

In order to test the motion compensation effect of the algorithm and the robustness to different signal strengths and characteristics, the measured data is divided into two steps. The experimental scenario in section 3.2.1 is a ground paved with striped bricks and a semi-metallic pipe below,which represents a non-uniform scene with high contrast.The experimental scenario in section 3.2.2 is the outer wall of the building, which is a uniform scene with low contrast.

#### 3.2.1 Verification of algorithm autofocus effect in non-uniform scene

In order to further illustrate the effectiveness of the PMEA algorithm, the actual measurement data is used for verification. The MEA and MCA algorithms are selected as the comparison algorithms for comparison.

Measured data from 77Ghz mounted on an eight-rotor UAV SAR system obtained. The system uses FM continuous wave signal with a bandwidth of 3.64Ghz.

**Fig 9:**
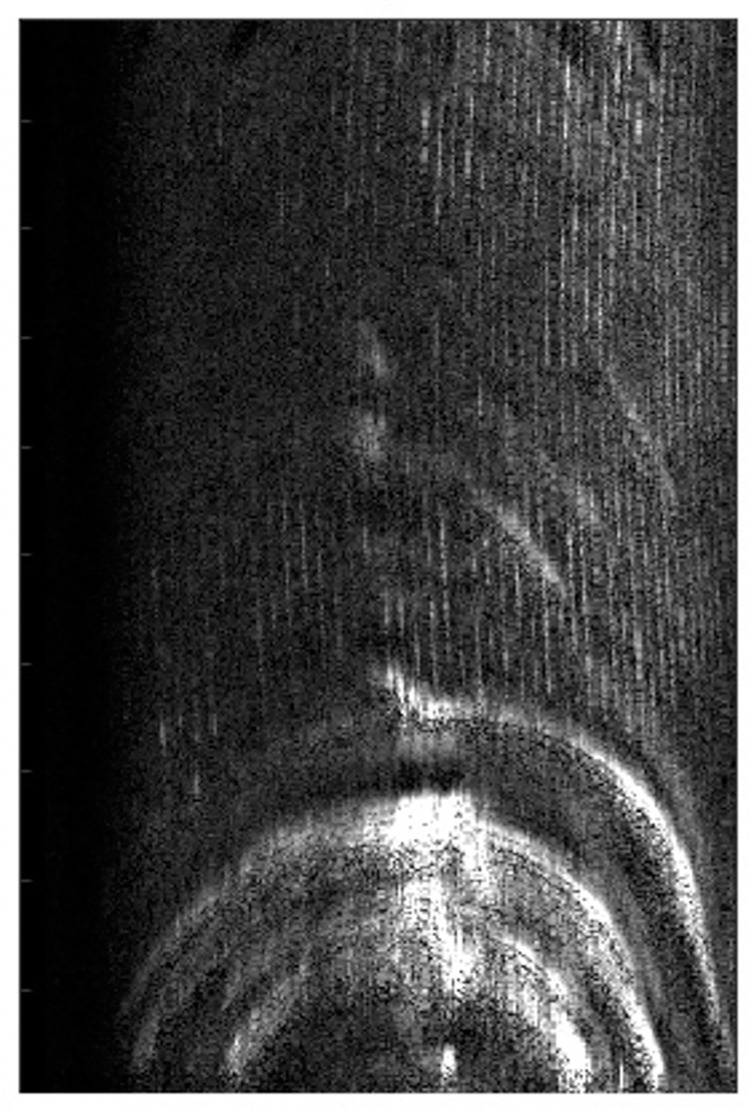
The real focused results.

**Fig 10:**
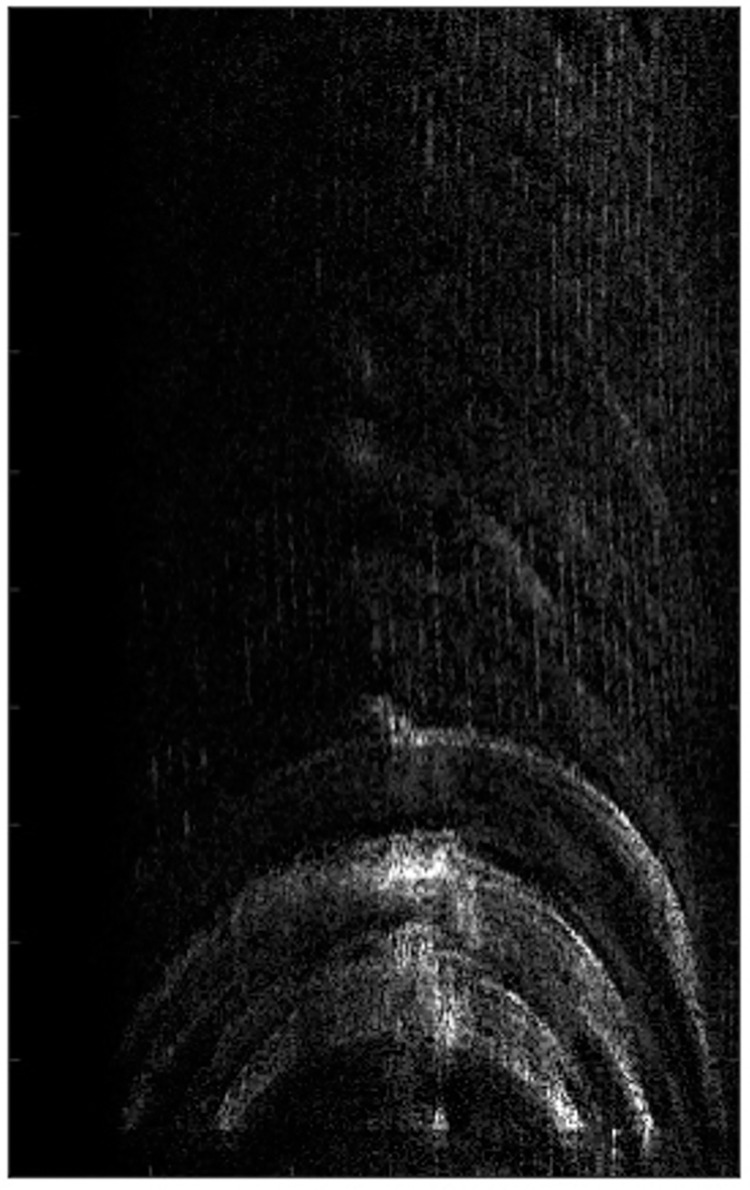
The real focused results.

**Fig 11:**
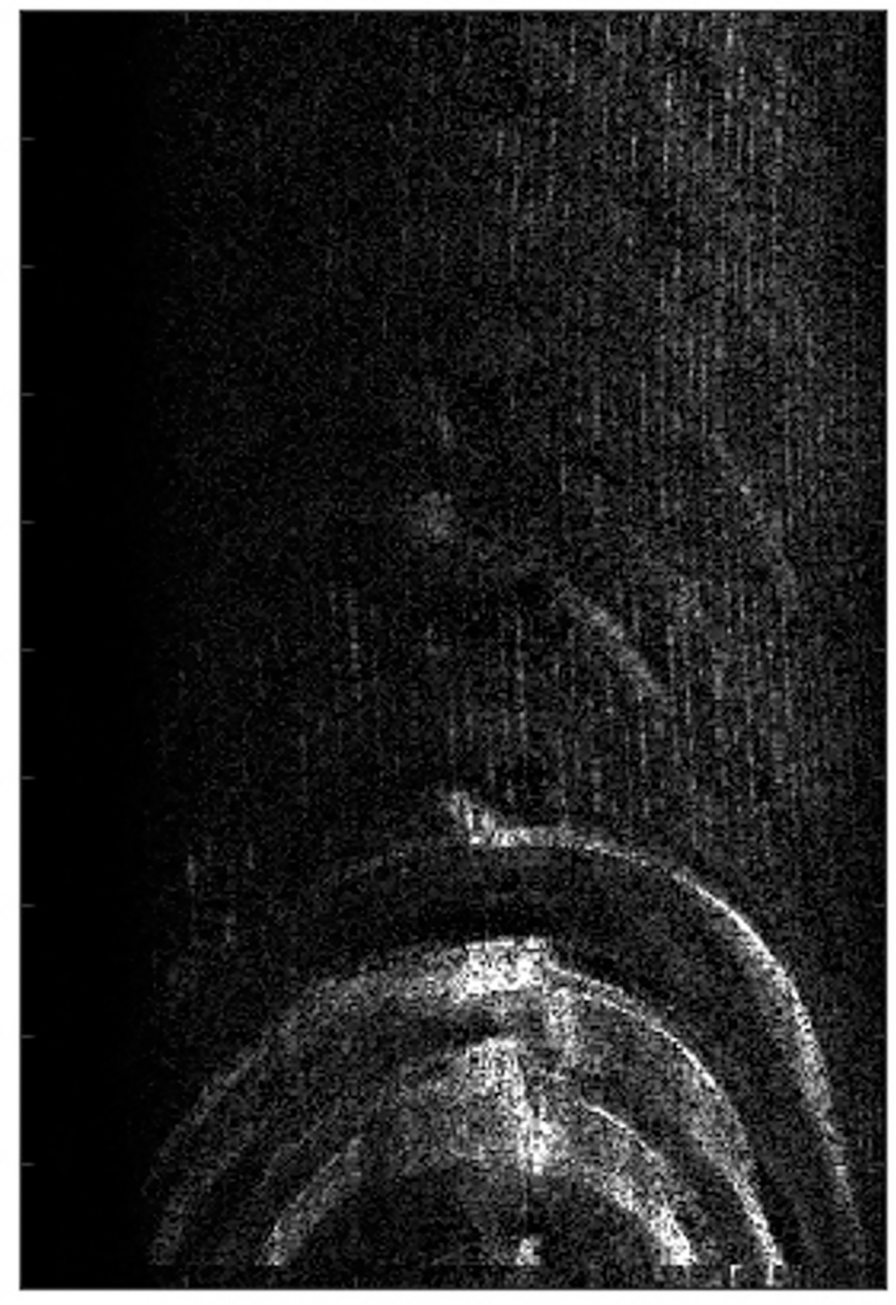
The real focused results.

It can be observed from the Figs 9–11 that when the MEA method and the MCA method with 5% stronger range cell are used, the image has obvious defocus.When the PMEA method is used, the focusing effect of the SAR image is better.

**Fig 12:**
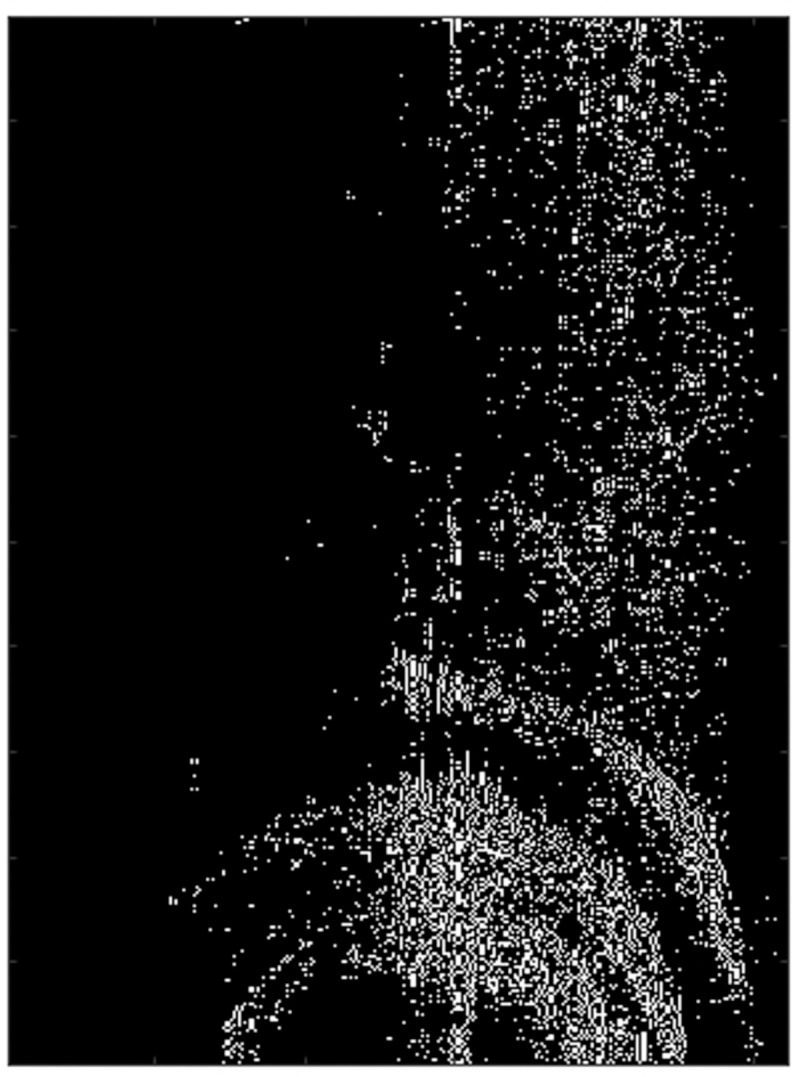
The result of extraction of SAR image feature points by Prewitt operator.

Fig 12 is the feature point map of the SAR image extracted by the Prewitt operator, and the bottom of the image is a semicircular metal tube. It can be observed that although the image has a certain defocus, the general outline of the image can still be extracted by using the Prewiit operator.

**Fig 13:**
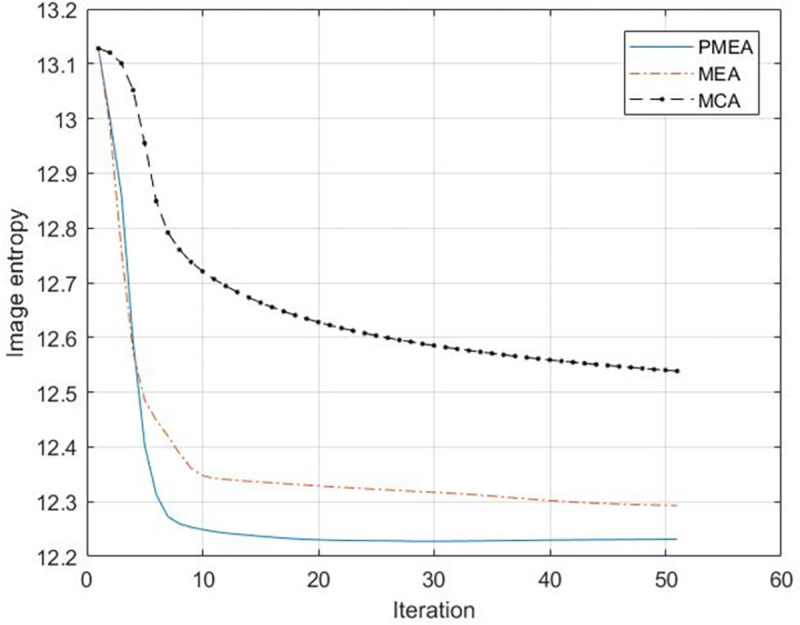
Results of entropy curve of real data.

The image entropy is used to compare each algorithm’s focusing effect. Fig 13 illustrates the entropy curve per algorithm after 50 iterations.The final entropy value of the image convergence and the average time of convergence is shown in Table 3, revealing that the SAR image utilizing PMEA for motion compensation has the best focusing effect. The remaining methods require more iterations and ultimately output inferior results than PMEA. Considering that the semicircular metal tube in the scene has strong reflection characteristics, the MCA and MEA methods bring a part of the range cell with strong energy into the iterative process, imposing the range cell substituted into the autofocus algorithm to be more concentrated in the range direction. Regarding the PMEA method, it considers the above problems and selects the range cell with a large number of feature points as input, making the distribution of the substituted range cell more uniform. On the other hand, the range cell with many feature points contains rich phase error information. Therefore, such range cell are selected as the input of the autofocus algorithm, effectively improving the motion compensation accuracy and the single iteration. Ultimately, the entropy value of the final image and the average convergence time is lower than the one of MEA and MCA.

**Table 3:** Comparison of measured data results.

	PMEA	MCA	MEA
**The final entropy value of the image**	12.23	12. 54	12.3
**Convergence Average Time (s)**	2.60	4.95	5.72

#### 3.2.2 Verification of algorithm autofocus effect in uniform scene

**Fig 14:**
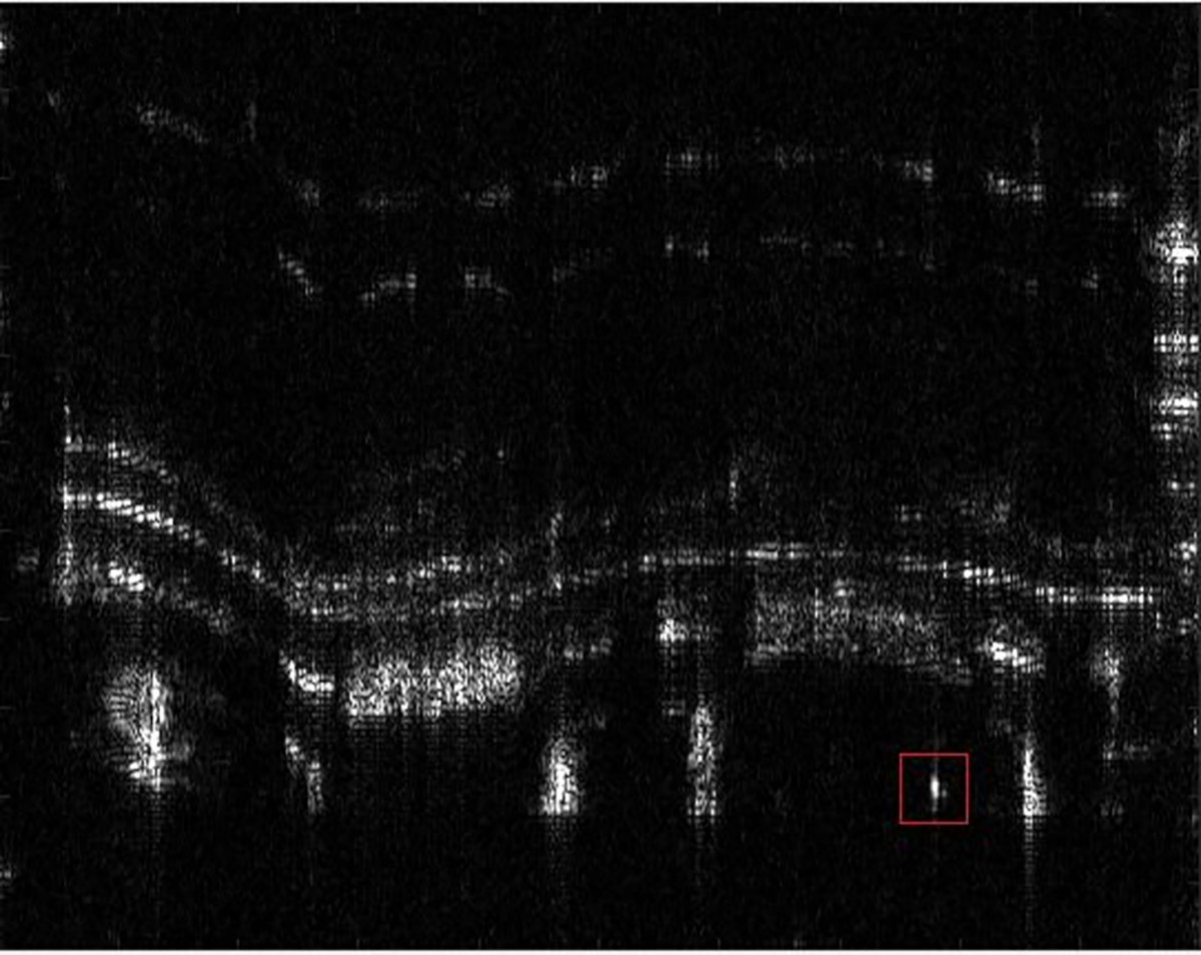
The real focused results.

**Fig 15:**
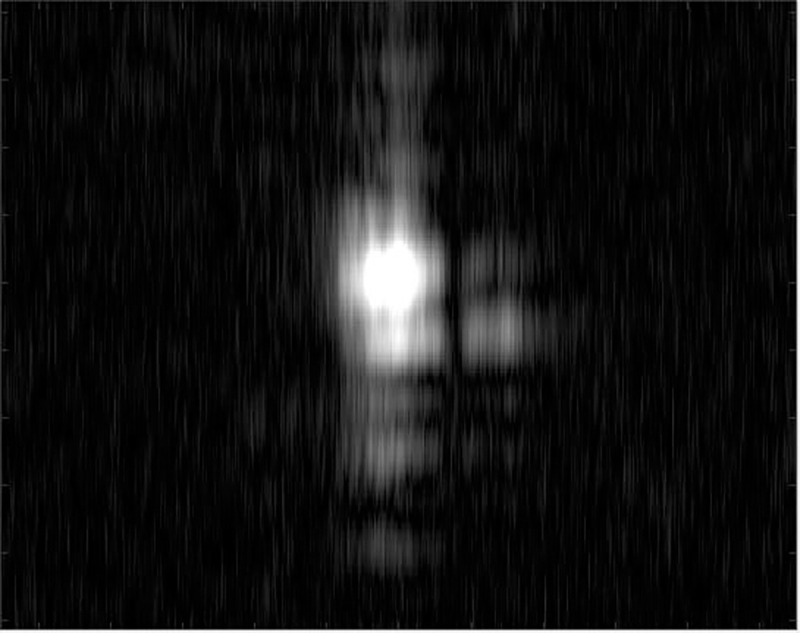
The real focused results.

**Fig 16:**
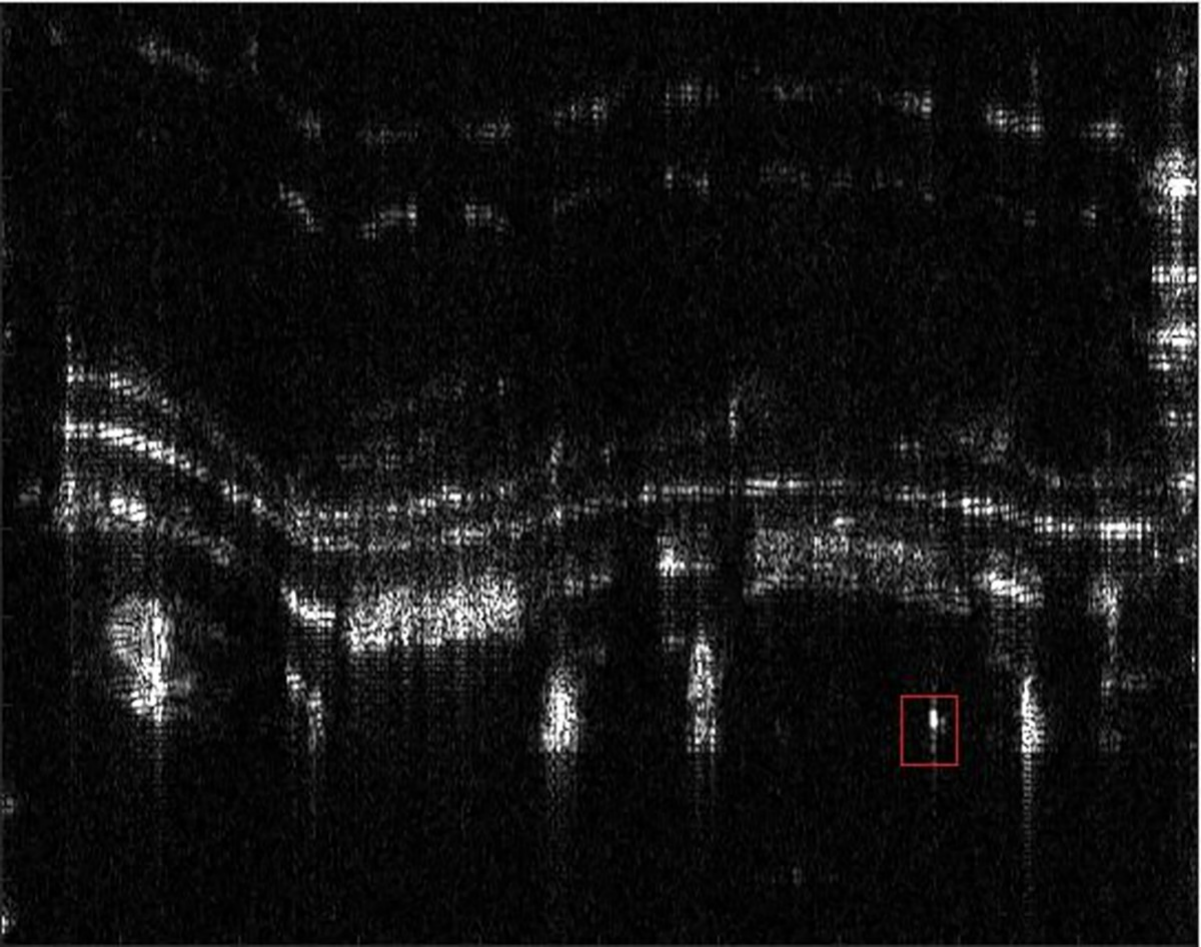
The real focused results.

**Fig 17:**
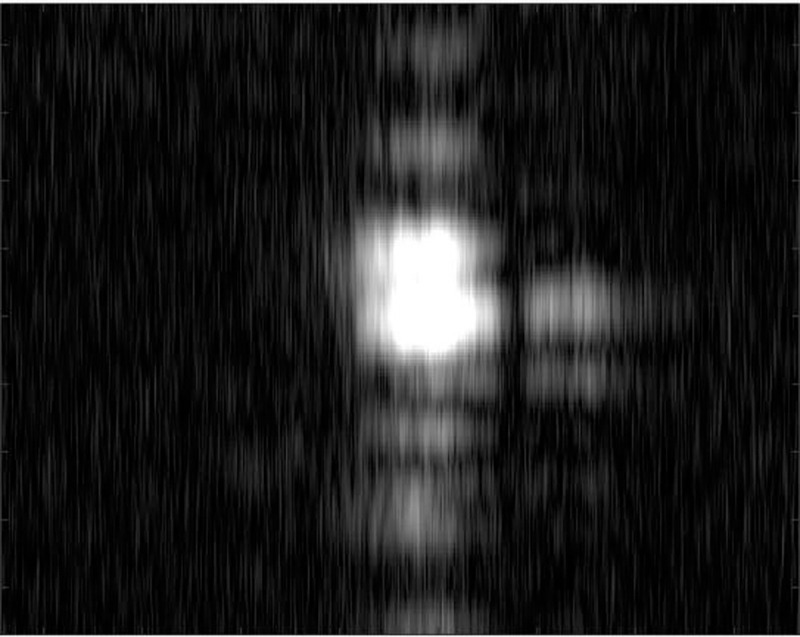
The real focused results.

In order to analyze the robustness of the algorithm to the focusing effect in various scenes, the author further verifies the autofocus effect of the PMEA algorithm by using the uniform scene with low contrast. The SAR experimental system is consistent with section 3.2.1. The experimental scene is the outer wall of the building, which is a uniform scene with low contrast. Selecting the PGA as the comparison algorithm for comparison. The focus result diagram of each algorithm and the method area in the red box are shown in Figs 14, 15, 16 and 17.

It can be seen from Figs 14–17 that the focusing effect of PMEA algorithm is better than that of PGA algorithm. For uniform scenes with low contrast, PMEA algorithm can still realize high-precision motion compensation of SAR images. The PGA algorithm selects the point with the largest amplitude in each range unit of SAR echo signal as the salient point to estimate the phase error. The quality of these salient points will greatly affect the accuracy of phase error estimation. The scene is a low contrast uniform scene. The signal behind the window of the PGA algorithm is greatly affected by the target background clutter so that the point with the largest amplitude is not an isolated point, which leads to the decline of the accuracy of the PGA algorithm, even the failure of autofocus.

PMEA algorithm is based on MEA algorithm and does not rely on strong scattering points. Therefore, it can still achieve high-precision motion compensation in low contrast scenes.

**Fig 18:**
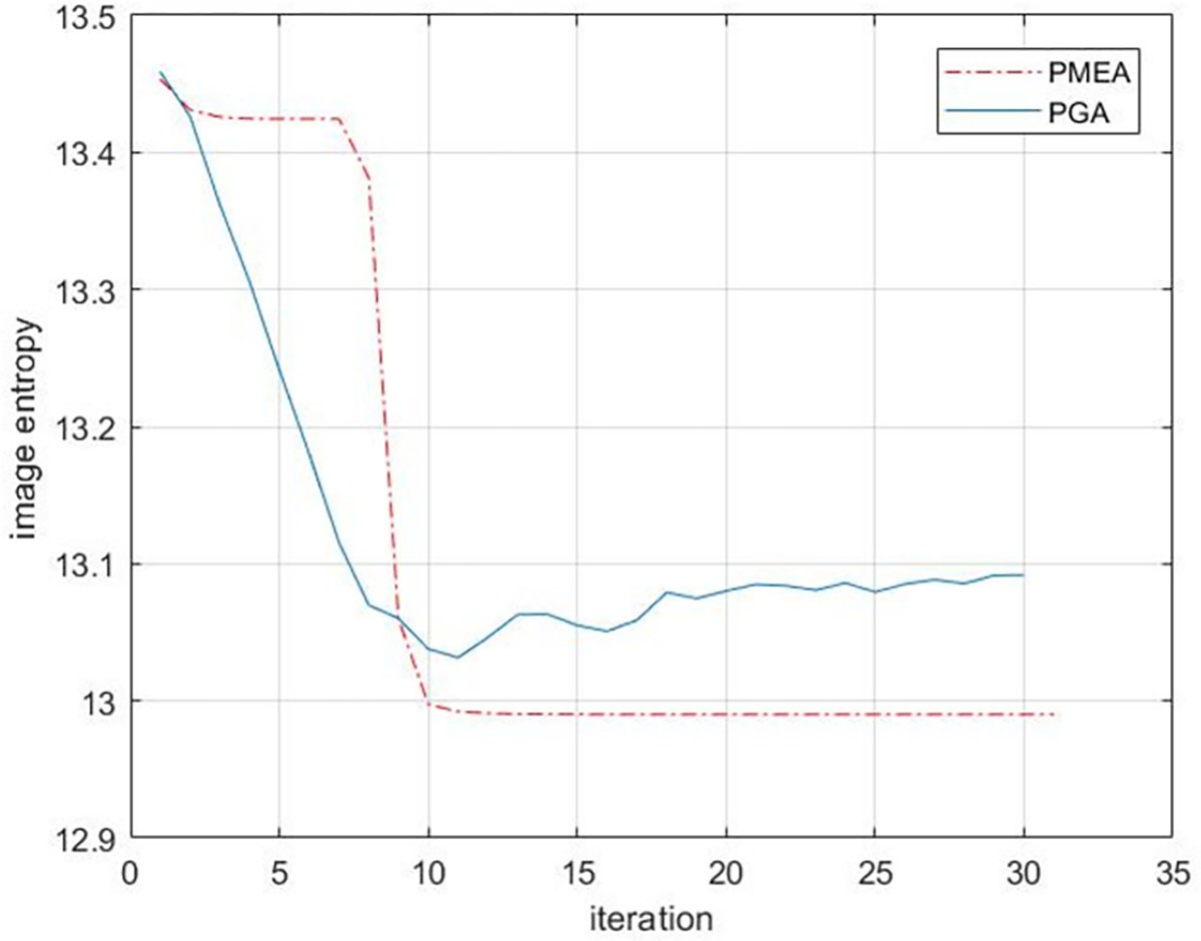
Results of entropy curve of real data.

The entropy of the image is still used to compare the focusing effect of each algorithm. In the 30 iterations of SAR image, the entropy curve corresponding to each algorithm is shown in Fig 18.

As shown in Fig 18, in the first 10 iterations of low contrast scenes, although the image entropy of the PGA algorithm decreases rapidly, the focus effect of the PGA algorithm is affected due to the lack of strong scattering points in the scene, and the algorithm falls into the wrong local optimal solution.Finally, the entropy cannot converge after 30 iterations. The PMEA algorithm shows good robustness. For low contrast scenes, the image entropy can still converge after about 10 iterations. And the final image entropy value is lower than that of PGA algorithm. Further, the PMEA algorithm selects the range cell containing 10% of the number of feature points of the image as the input of the algorithm, which greatly reduces the calculation amount of the algorithm and improves the operation efficiency of the algorithm. On the other hand, the range cell containing more feature points in the SAR image contains rich phase error information, so the PMEA algorithm also maintains high focus accuracy.

## 4. Conclusion

This paper develops an improved autofocus method based on the Prewitt operator. Our method first uses the Prewitt operator to obtain the feature points of the SAR image and then selects a part of the range cell with many feature points as the input to the autofocus algorithm. Simulation and experimental results prove that the proposed method improves the MEA’s computational efficiency and motion error compensation accuracy. Compared with the classic autofocus algorithm, The autofocus algorithm proposed in this paper has better motion compensation effect and higher computational efficiency

## Supporting information

S1 Data (ZIP)
